# Optical pulling at macroscopic distances

**DOI:** 10.1126/sciadv.aau7814

**Published:** 2019-03-29

**Authors:** Xiao Li, Jun Chen, Zhifang Lin, Jack Ng

**Affiliations:** 1 Department of Physics, Hong Kong Baptist University, Hong Kong, China.; 2 Institute of Theoretical Physics and Collaborative Innovation Center of Extreme Optics, Shanxi University, Shanxi, China.; 3 State Key Laboratory of Surface Physics, Key Laboratory of Micro and Nano Photonic Structures, and Department of Physics, Fudan University, Shanghai, China.; 4 Collaborative Innovation Center of Advanced Microstructures, Nanjing University, Nanjing, China.; 5 Institute of Computational and Theoretical Studies, Hong Kong Baptist University, Hong Kong, China.

## Abstract

Optical tractor beams, proposed in 2011 and experimentally demonstrated soon after, offer the ability to pull particles against light propagation. It has attracted much research and public interest. Yet, its limited microscopic-scale range severely restricts its applicability. The dilemma is that a long-range Bessel beam, the most accessible beam for optical traction, has a small half-cone angle, θ_0_, making pulling difficult. Here, by simultaneously using several novel and compatible mechanisms, including transverse isotropy, Snell’s law, antireflection coatings (or impedance-matched metamaterials), and light interference, we overcome this dilemma and achieve long-range optical pulling at θ_0_ ≈ 1°. The range is estimated to be 14 cm when using ~1 W of laser power. Thus, macroscopic optical pulling can be realized in a medium or in a vacuum, with good tolerance of the half-cone angle and the frequency of the light.

## Teaser

A cocktail of independent yet compatible mechanisms pull an object from far with light.

## INTRODUCTION

An optical force is well known for its ability to “trap” or “propel” microscopic particles (*1*–*26*). A counterintuitive phenomenon was discovered in 2011 (*27*–*31*): By redirecting the incident photons forward, the light-induced Lorentz force can pull distant objects backward against the propagation of light. This phenomenon, now termed the optical pulling force (OPF) or optical tractor beam (OTB), is emerging as a reality in laboratories (*32*, *33*) and offering new functionality in optical micromanipulation. Yet, its short micrometer-scale working range remains an obstacle that limits its practical applications.

**Fig. 1:**
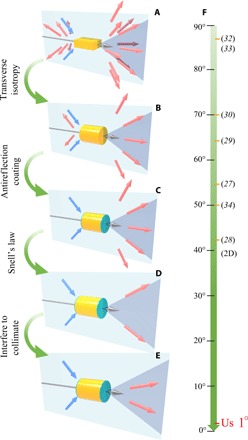
Illustration of the series of mechanisms applied, in turn, to achieve the OPF. The yellow particle is illuminated by a transversely isotropic Bessel beam, which propagates in the direction of the gray arrow, and its constituting *k* vectors lie on the cone formed by the revolution of the blue arrows about the partial axis. The green layer represents the ARC, and the semitransparent black cone is the pulling cone. In (**A**), the scattered fields are represented by the pink arrows. In (**B**) to (**E**), the scattered fields are represented by the cone formed by the revolution of the red arrows about the partial axis. (A) Scattering by a general particle. Light is scattered everywhere. (B) Introducing transverse isotropy to eliminate the diffraction in the azimuthal direction. (C) Introducing ARC to eliminate reflection. (D) Snell’s law approximately aligns the scattered field to the edge of the pulling cone. (E) The diffracted light is collimated by the interference. (**F**) Half-cone angles achieved in the literature.

A photon propagating in the direction θ_0_ and elastically scattered into angle θ by a particle will transfer to the particle a forward momentum of

$$\Delta p_{z}=\hbar k(\text{cos}\theta_{0}-\text{cos}\theta )$$ (1)

where the first and second terms are the initial and final momentum of the photon, respectively. The particle first intercepts the photon, resulting in an extinction force, which is given by the first term in Eq. 1. It then re-emits the photon, leading to a recoil force, given by the second term (*29*). Because the extinction force is positively definite, to generate the OPF, the recoil force must be negative and stronger than the extinction force. Equation 1 contributes to pulling when θ < θ_0_ and pushing when θ > θ_0_. Inducing the OPF is equivalent to squeezing most of the scattered light into a solid cone defined by θ < θ_0_, which is hereafter referred to as the pulling cone. Thus, in general, a small θ_0_ is unfavorable for the OPF. In the literature, the minimum θ_0_ reported for the OPF is ~50° in simulations using a Bessel beam (*34*) and ~86° in experiments that essentially used a pair of plane waves (*32*). In the hypothetical case of a “2D” (two-dimensional) system, simulations found the OPF at ~42° (*28*). The values of θ_0_ achieved in the literature are schematically summarized in Fig. 1F.

Ideal Bessel beams have an infinite cross section and energy. Therefore, they do not exist in reality. One could produce an approximate Bessel beam, which is nondiffracting over a distance long compared to the Rayleigh range of a Gaussian beam. The range of a Bessel beam, as produced by an axicon, is roughly given by (*35*)

$$\text{Range}(\theta_{0})\approx \omega_{0}/\text{tan}(\theta_{0})$$ (2)

where ω_0_ is the waist of the incident beam that illuminates the input side of the axicon, which is assumed to be 2.5 mm, as in the experiment of (*36*). To extend its range, it is impractical to adopt a very large ω_0_, which also requires a large axicon. One may then take the alternative route of decreasing θ_0_. However, this causes a practical dilemma in optical pulling: Although the range of the beam is short for large θ_0_ (*35*), it is difficult to achieve the OPF if θ_0_ is small (*29*). We note that a Bessel beam or other propagation invariant beam can also be produced by other approaches (*35*, *37*–*39*).

Here, we completely overcome this dilemma in terms of the range and half-cone angle by enabling OPF at small θ_0_ down to 1^o^, which is approximately two orders of magnitude smaller than the previously reported angle of ~50° in 3D (*34*), as illustrated in Fig. 1F. We achieved this by eliminating diffraction in the azimuthal direction by transverse isotropy (Fig. 1, A and B), eliminating reflection by using antireflection coatings (ARCs) (Fig. 1C), restricting the diffracted wave to propagate near the edge of the pulling cone by using cylindrical geometry to enforce Snell’s law (Fig. 1D), and finally collimating the diffracted light by interference to induce OPF (Fig. 1E). For θ_0_ = 1^o^, the range of the Bessel beam could be >14 cm according to Eq. 2. We note that, to produce a long-range Bessel beam with θ_0_ = 1^o^, the available laser power is also a limiting factor. The total power for the central ring of the Bessel beam is 0.3 W for θ_0_ = 1^o^. The total power needed will be of this order of magnitude. Other than this constraint in power, however, long-range pulling is not forbidden by physical laws.

Other interesting optical forces, such as optical lift (*40*) and lateral forces perpendicular to the light propagation direction (*41*–*45*), have also been reported. Tractor beams not based solely on the Lorentz force are also of immense interest. They have been proposed by using, for example, photophoretic forces (*46*, *47*), acoustic forces (*48*–*50*), and even matter waves (*51*), in addition to optical beams (*52*–*59*). The tractor beam is a highly counterintuitive construct, although it violates no fundamental physical law. Most tractor beams, even those relying exclusively on lasers, require a medium to function, such as air or water. Some, such as an acoustic tractor beam, require a medium for the wave itself to propagate. Others, such as the photophoretic tractor beam, work by light-induced thermal forces that cannot exist without a medium. Here, we stress that the OTB we designed operates on the OPF alone, and it can work in a vacuum. This is an important step toward remote sampling in a vacuum, which is desirable in, for example, space exploration; light can both push and pull a handle to collect samples from a remote site.

## RESULTS

### Pulling enhanced by transverse isotropy

Consider a hypothetical 2D system that consists of a slab and a beam that are translationally invariant along one direction. By symmetry, light diffraction is limited to within the scattering plane, so the degrees of freedom for the light to diffract are reduced from 2 to 1. Then, among all possible scattering angles, a fraction of θ_0_/π contributes to pulling, which is significantly better than the fraction of sin^2^ θ_0_/2 in a general 3D system at small θ_0_. One may thus intuitively expect the OPF to be much more achievable in 2D in general. At θ_0_ = 1^o^, the pulling cone spans 5.6 × 10^−3^ out of all solid angles, which is more than 70 times larger than that of a 3D system, which is 7.6 × 10^−5^. However, the fraction is very small in either case; thus, pulling at θ_0_ ≈ 1^o^ is not easy at all. Moreover, 2D systems, if not somewhat fictive, are very long in one dimension; thus, they are quite heavy and difficult to manipulated by light.

Here, we note that a system with transverse isotropy shares the advantages of a 2D system but not its disadvantages. Because a system consists of a particle and a beam that have transverse isotropy, the Poynting vectors of the incident and scattered fields have no azimuthal component; therefore, diffraction in the azimuthal direction is forbidden. Consequently, light propagating in the plane defined by a constant ϕ remains on that plane after being scattered. This, in a sense, mimics 2D diffraction.

The OPF is typically realized using a propagation-invariant beam, which, ideally, does not diffract as the beam propagates. Here, if not otherwise specified, we use the *m* = 0 azimuthally polarized Bessel beam

$$E=-E_{0}i\;\text{exp}(ik_{0}\;\text{cos}\;\theta_{0}z)\frac{J_{0}\prime (k_{0}\;\text{sin}\;\theta_{0}\rho )}{\text{sin}\;\theta_{0}}\hat{\phi }$$ (3)

where *k*_0_ is the background wave number, (ρ, ϕ, *z*) are the cylindrical coordinates, $J_{0}\prime$ is the derivative of the zeroth-order Bessel function with respect to its argument, and the fixed half-cone angle θ_0_ denotes the angle between the beam propagation direction ($\hat{z}$) and the wave vectors of the Fourier components of Eq. 3. Throughout this paper, unless explicitly stated, the incident wavelength is fixed at 532 nm. The normalization of the Bessel beam’s intensity is described in Materials and Methods.

**Fig. 2:**
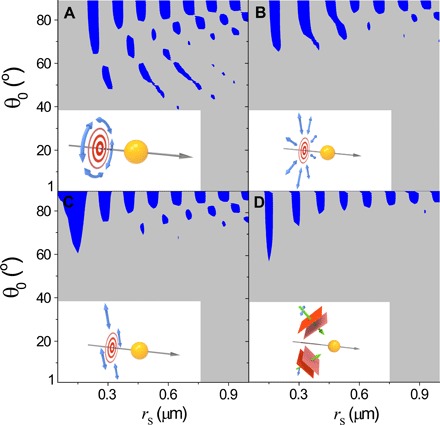
Pulling enhanced by transversely isotropic beam. The blue color indicates the phase space region where the OPF exists. The insets are schematic illustrations of a sphere (*n*_p_ = 1.6) in water (*n*_b_ = 1.33) illuminated by a different beam. (**A**) *m* = 0 azimuthally polarized Bessel beam (transversely isotropic) (see Eq. 3). (**B**) *m* = 0 radially polarized Bessel beam (transversely isotropic) (see Eq. 4). (**C**) *m* = 0 linearly polarized Bessel beam (nontransversely isotropic) (see Eq. 5). (**D**) Propagation-invariant beam formed by a pair of plane waves (nontransversely isotropic) (see Eq. 6).

Figure 2 plots the OPF acting on a spherical particle illuminated by different concentric beams, including the transversely isotropic *m* = 0 azimuthally polarized Bessel beam given in Eq. 3 (Fig. 2A), a transversely isotropic *m* = 0 radially polarized Bessel beam (Fig. 2B)

$$E=E_{0}\;\text{exp}(ik_{0}\;\text{cos}\;\theta_{0}z)(i\frac{\text{cos}\;\theta_{0}J_{0}\prime (k_{0}\;\text{sin}\;\theta_{0}\rho )}{\text{sin}\;\theta_{0}}\hat{\rho }+J_{0}\prime (k_{0}\;\text{sin}\;\theta_{0}\rho )\hat{z})$$ (4)

an *m* = 0 linearly polarized Bessel beam (Fig. 2C)

$$E=E_{0}\;\text{exp}(ik_{0}\;\text{cos}\;\theta_{0}z)(\text{cos}\;\theta_{0}J_{0}(k_{0}\;\text{sin}\;\theta_{0}\rho )\hat{y}-i\frac{y}{\rho }\text{sin}\;\theta_{0}J_{0}\prime (k_{0}\;\text{sin}\;\theta_{0}\rho )\hat{z})$$ (5)

and a pair of copropagating plane waves (Fig. 2D)

$$E=E_{0}\hat{x}(e^{ik_{0}\;\text{sin}\;\theta_{0}y}+e^{-ik_{0}\;\text{sin}\;\theta_{0}y})e^{ik_{0}\;\text{cos}\;\theta_{0}z}$$ (6)

In terms of inducing the OPF, the azimuthally polarized beam given in Eq. 3, which is transversely isotropic, outperformed all others. The radially polarized beam was also transversely isotropic, but it was still expected to be less effective in pulling. The electromagnetic boundary conditions required the electromagnetic fields for the azimuthal polarization to be continuous across the particle boundary, resulting in less reflection, and thus, it had a stronger OPF. However, the electric field for the radial polarization was discontinuous, leading to stronger reflection; thus, it had a weaker OPF. That is, the difference in performance between the radial and azimuthal polarization was due to the asymmetry in permittivity and permeability distribution. We note that Fig. 2 also suggests that OPF depends not only on the type of beam but also on its polarization.

**Fig. 3:**
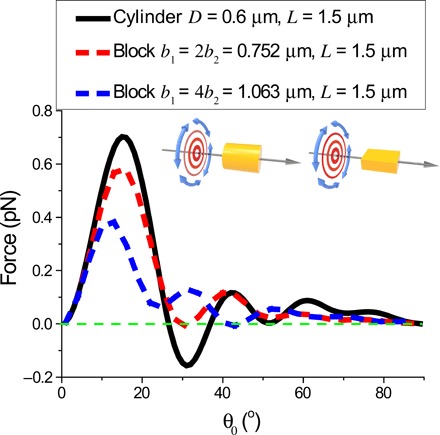
Pulling enhanced by transversely isotropic particle. The OPF induced by an *m* = 0 azimuthally polarized Bessel beam (transversely isotropic) acting on a transversely isotropic circular cylinder (solid line) with diameter *D* and length *L* and nontransversely isotropic rectangular blocks (dashed line) with different aspect ratios (*b*_1_/*b*_2_) and length *L*. Inset: Schematic illustration of the geometries.

Figure 3 compares the optical force acting on a dielectric cylinder and two rectangular dielectric blocks with different aspect ratios. The incident beam was the Bessel beam given in Eq. 3. All particles had the same volume and length and were made from the same material. The OPF diminished with increasing aspect ratio of the rectangular blocks (red and blue dashed curves) as the particle shape deviated from transverse isotropy. A highly symmetric system places more restrictions on the light propagation; therefore, the light has a lesser degree of freedom to diffract, which is favorable for the OPF where strong forward scattering is required.

Figures 2 and 3 highlight the importance of transverse isotropy in optical pulling. Although the magnitude of the pulling force was smaller than that of the pushing force, they were of the same order of magnitude. We also calculated the OPF acting on a spheroid (see section S1) induced by the Bessel beam given in Eq. 3. Its performance in the OPF was even better than that of a bare cylinder. The reflection from the flat ends of the cylinder was directly backward, generating strong forward forces, whereas that of the spheroid did not. The good performance of the spheroid further confirms the important role of transverse isotropy in optical pulling. For reasons to be discussed later, we still preferred working with the cylinder.

### Pulling enhanced by Snell’s law

**Fig. 4:**
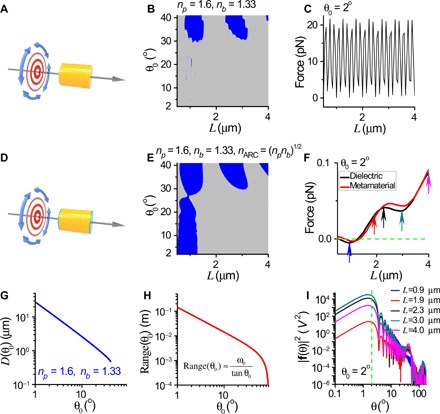
OPF acting on a dielectric cylinder of diameter *D* and length *L*. Schematic illustration without ARC (**A**) and with ARC (**D**). (**B** and **E**) Phase space plots for the OPF acting on the dielectric cylinders without [see (A)] and with [see (D)] ARC, respectively, with *D*(θ_0_) shown in (G). Blue and gray regions indicate pulling and pushing forces, respectively. (**C**) Optical forces acting on the cylinder shown in (A) versus the length of the cylinder *L* when θ_0_ = 2^o^. (**F**) Black: Optical forces acting on the cylinder shown in (D) versus the length of the cylinder *L* when θ_0_ = 2^o^. Red: The OPF acting on a metamaterial cylinder made of ε_r_ = *n*_p_*n*_b_ and μ_r_ = *n*_p_/*n*_b_. (**G**) Diameter *D*(θ_0_) of the cylinder at which the FWM is excited. (**H**) Range of the Bessel beam versus θ_0_. (**I**) Angular distribution of the scattered fields for pulling (blue) and pushing (others) forces, marked by arrows in (F) with corresponding colors.

We went beyond transverse isotropy and considered a dielectric microcylinder of diameter *D* and length *L*, coated without (Fig. 4A) and with (Fig. 4D) ARC. To induce the OPF at a small θ_0_, the particle must squeeze most of the scattered light into the tiny pulling cone. Apparently, this would not be possible if the scattering was strong, as light would be scattered everywhere. In Fig. 4B, the phase space region with the OPF is highlighted in blue for a bare glass cylinder without ARC in water and illuminated by the Bessel beam given in Eq. 3. Because the microcylinder had straight parallel sides and flat ends, light impinging on it reflected and diffracted approximately according to Snell’s law but with some slight deviation due to diffraction by the particle with finite size and finite curvature. Accordingly, by Snell’s law, all light was scattered into an angle near the edge of the pulling cone, i.e., θ = θ_0_ + Δθ, where the small Δθ was induced by diffraction. It turns out that if one further excites the fundamental waveguide mode (FWM) by using a particle with an appropriate size, then Δθ < 0, which induces the OPF, as explained in the next section. Here, at each θ_0_, we chose a diameter *D* such that the FWM was excited. The relationship between *D* and θ_0_ is plotted in Fig. 4G, which can be written down analytically for small θ_0_

$$D(\theta_{0})\approx 2z_{1}/k_{b}\theta_{0}$$ (7)

Here, *z*_1_ is the first zero of the first-order Bessel function. Inducing the OPF at small θ_0_ is possible only with a large *D* owing to the diffraction limit. Further details on the FWM and the derivation of Eq. 7 can be found in section S2. We must stress that the excitation of the FWM is by no means a requirement for the OPF, although it can be an advantage sometimes. The FWM can be effectively excited only for θ_0_ less than some critical value, and the OPF for θ_0_ outside this range is not plotted on the figures. The OPF exists for θ_0_ > 30^o^. A larger θ_0_ has a larger pulling cone; therefore, it is more likely for the scattered light to induce the OPF. When θ_0_ is small (<30°), the OPF almost disappears completely because of the strong reflection by the flat end of the cylinder. The optical force versus *L* when θ_0_ = 2^o^ is plotted in Fig. 4C. We observed some periodically spaced peaks, which can be attributed to the Fabry-Perot resonances.

### Pulling enhanced by ARCs

To further improve the OPF, we noted that backscattering generated a strong forward force. For particles with flat ends, such as a cylinder, ARCs may be applied on its ends to reduce or even completely eliminate reflection. We noted that ARC was also adopted in (*28*, *60*–*62*) to improve the performance of the optical force and the OPF. Our ARCs were characterized by a refractive index

$$n_{\text{ARC}}=\sqrt{n_{p}n_{b}}$$ (8)

and a thickness

$$d(\theta_{0})=\frac{\lambda \;\text{cos}\;\theta_{\text{ARC}}}{4n_{\text{ARC}}-4n_{b}\;\text{sin}\;\theta_{0}\;\text{sin}\;\theta_{\text{ARC}}}$$ (9)

where θ_ARC_ = sin^−1^(*n*_b_ sin θ_0_/*n*_ARC_). The coating described by Eqs. 8 and 9 ensured that the reflected waves from the two boundaries of the coating would interfere destructively. The reflection was completely eliminated at normal incidence and partially eliminated at oblique incidence. A discussion on the tolerance and robustness of OPF with respect to the ARC thickness and its nonuniformity is given in section S5. That is, in typical cases, the OPF survives for nonuniformity in ARC thickness up to several tens of nanometers. The configuration considered in Fig. 4E is the ARC-coated version of Fig. 4B, and its geometry is illustrated in Fig. 4D. After coating, the phase space area with the OPF expanded considerably, especially for small *L* and small θ_0_, where the ARC worked best. Figure 4F plots the optical force versus *L* when θ_0_ = 2^o^ for the ARC-coated cylinder. The Fabry-Perot resonances observed in Fig. 4C were eliminated by the ARC as the light could no longer bounce back and forth. The reduced reflection also uniformly shifted the force toward negative values as compared to the uncoated case. We observe two dips at *L* = 0.9 μm and *L* = 2.9 μm in Fig. 4F. The force oscillated with *L* with a period Δ = λ/(*n*_p_ − *n*_b_) ≈ 2 μm owing to the interference structure (*63*). There was no OPF for long particles when θ_0_ was small. Consider the light rays that go into the cylinder from the curved side surface and go out through the flat end. According to Snell’s law, if these light rays enter with an angle of θ_0_ = 2^o^, they will exit with an angle of θ_out_ ≈ 42^o^, which generates strong forward forces. The longer the particle is, the more it is affected and therefore the less likely for the OPF to exist. The angular distributions of the scattered field when θ_0_ = 2^o^ for different cylinder lengths are shown in Fig. 4I and marked as different colored arrows in Fig. 4F. First, we note that the peaks of all the scattered fields were located nicely inside the pulling cone, which was due to the collimation effect induced by interference, to be discussed in the next section. Second, there was a non-negligible scattered field at the direction θ ≈ 42^o^, which was due to the light entering from the curved side.

We remark that a cylinder made from impedance-matched metamaterials, such as those with ε_r_ = *n*_p_*n*_b_ and μ_r_ = *n*_p_/*n*_b_ (*64*–*66*), was able to replace the ARC-coated cylinder. This outcome was equally good, as shown by the red curve in Fig. 4F.

### Pulling enhanced by collimation due to interference

The angular distribution of the scattered light plotted in Fig. 4I shows a scattering peak located within θ < 2^o^. The exit end of the cylinder can be approximately treated as a circular aperture (*67*), where the plane wave–like guided light diffracts upon exit. Because Snell’s law is approximately satisfied for the flat ends and the straight parallel curved sides of the microcylinder, the field scattered by an ARC cylinder approximately preserves the light propagation angle such that θ ≈ θ_0_ for nearly all scattered light. However, the interference between different Fourier components of the light induces a shift in θ toward 0°. To see this, consider a circular aperture (i.e., the exit end of the cylinder) illuminated uniformly by a plane wave propagating along (θ_0_, ϕ_0_ = 0) and polarized along $\hat{\phi }=\hat{y}$; on the ϕ = 0 plane (i.e., *xz*-alnbe), the diffracted wave is (*67*, *68*)

$$E_{\text{diff},+\theta_{0}}(r,\theta ,\phi =0)=\hat{y}\frac{ie^{ikr}}{r}a^{2}\text{cos}\;\theta E_{0}\frac{J_{1}(ka(\text{sin}\;\theta -\text{sin}\;\theta_{0}))}{ka(\text{sin}\;\theta -\text{sin}\;\theta_{0})}$$ (10)

where *a* = *D*(θ_0_)/2. According to Eq. 10, for a single plane wave, the angular distribution peaks at θ ≈ θ_0_, as expected from Snell’s law. However, if one considers adding a second plane wave, which is identical to the first one except ϕ = π, the total diffracted field will be

$$\begin{array}{c} E_{\text{diff}}(r,\theta ,\phi =0)=E_{\text{diff},+\theta_{0}}(r,\theta ,\phi =0)+E_{\text{diff},-\theta_{0}}(r,\theta ,\phi =0)\\ =\hat{y}\frac{ie^{ikr}}{r}a^{2}\text{cos}\;\theta E_{0}(\frac{J_{1}(ka(\text{sin}\;\theta -\text{sin}\;\theta_{0}))}{ka(\text{sin}\;\theta -\text{sin}\;\theta_{0})}-\frac{J_{1}(ka(\text{sin}\;\theta +\text{sin}\;\theta_{0}))}{ka(\text{sin}\;\theta +\text{sin}\;\theta_{0})}) \end{array}$$ (11)

**Fig. 5:**
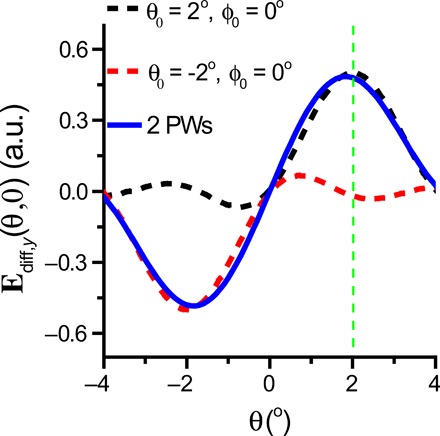
Pulling force induced by diffraction. The diffracted field emerging from a circular aperture of diameter *D*(θ_0_ = 2^o^) = 14 μm illuminated by one (Eq. 10) or two (Eq. 11) plane waves. The constructive interference between the two plane waves (PWs) at −2° < θ < 2° collimates the beam such that |θ| < 2°, which induces the OPF. a.u., arbitrary units.

One can observe from Fig. 5 that the peak scattering angle (θ_SP_) shifts from θ_SP_ = θ_0_ to θ_SP_ < θ_0_ after interference. Consider the black line shown in Fig. 5, which shows the scattered field distribution for a plane wave with θ_0_ = 2^o^. The angular distribution of the corresponding scattered field also peaks at θ = 2^o^. However, after it interferes with the red line induced by the second plane wave with θ_0_ = −2^o^, because the central maximum of the diffracted light is twice as wide as the higher-order resonances, the θ < 2^o^ side of the black curve interferes constructively, whereas the θ > 2^o^ side interferes destructively. As a result, the peak θ_SP_ shifts into the pulling cone where |θ| < 2^o^, which collimates the wave and contributes to OPF. We note that the shifting of θ_SP_ by our model with two plane waves was ~0.3°, whereas the shift for the Bessel beam shown in Fig. 4I was ~0.5°. The plane waves were not transversely isotropic, so their performance was not expected to be as good as that of the Bessel beam. More details about the interference of the diffracted fields can be found in section S3.

### Robustness of OPF

**Fig. 6:**
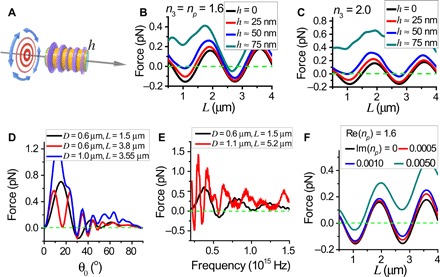
Robustness of the OPF. The incident beam is the azimuthally polarized Bessel beam (Eq. 3). (**A**) Schematic illustration for a circular cylinder (yellow) coated with ARC (green) and irregular additional structures (purple) with a maximum thickness *h* (the relative heights of the additional structure are drawn to scale). The incident beam is the *m* = 0 azimuthally polarized Bessel beam with θ_0_ = 35^o^, and *D* is chosen such that FWM is excited. (**B**) Optical force for the structure shown in (A) at various *h* when *n*_3_ = 1.6. Here, *n*_3_ is the refractive index for the additional structures. (**C**) Optical force for the structure shown in (A) at various *h* when *n*_3_ = 2.0. (**D**) Optical force for three bare cylinders of different sizes versus θ_0_. (**E**) Optical force for two bare cylinders of different size versus light frequency (θ_0_ = 30^o^). (**F**) Optical force versus length for ARC-coated cylinders made of materials with different absorption levels (θ_0_ = 35^o^).

The excellent performance of the OPF was based on several independent yet compatible mechanisms working in tandem. First, the transverse isotropy, cylindrical geometry of the system, and ARC ensured that θ ≈ θ_0_; then, the interference of FWM collimated the beam, which induced the OPF, as delineated in Fig. 1 (A to E). Figure 6A shows a transversely isotropic but otherwise irregular particle illuminated by the Bessel beam given in Eq. 3. It consists of a cylindrical core (yellow), plus an additional subwavelength-sized structure whose size was less than 100 nm (purple), and ARC (green) covering the flat ends of the cylinder but not the additional structure. Figure 6 (B and C) plots the forces when the refractive indices of the additional structures (purple) are *n*_3_ = *n*_p_ = 1.6 and *n*_3_ = 2.0, respectively. The OPF tolerated the existence of the additional structures when *n*_3_ = *n*_p_ = 1.6. For *n*_3_ = 2.0, the OPF was weakened. In all cases, the weakening of the OPF was mainly due to the reflection by the additional structures.

We also tested the tolerance of the OPF against θ_0_, incident wavelength, and material absorption, as shown in Fig. 6 (D to F). The nonresonance-based OPF had good tolerance in the converging angle, which was ± 5^o^ about the central angle at θ_0_ = 30^o^, as shown in Fig. 6D. In addition, the OPF had a relatively broad frequency band (~50- to 100-nm bandwidth), as shown in Fig. 6E. The optical forces versus *L* at different absorption levels in the dielectric constant of the particle were also investigated, and the results are shown in Fig. 6F. When Im(*n*_*p*_) = 0.0005, the absorption was noticeable only for particles longer than 4 μm because the longer the particle is, the more it absorbs. This degrades the OPF in two ways. First, it directly induces forward forces as light momentum is absorbed. Second, it indirectly reduces the amount of light available for generating a recoil force. When Im(*n*_*p*_) = 0.0050, OPF was observed only for short particles with lengths under ~ 1.3 μm. We remark that unlike high dielectrics, which are often associated with absorption, lower dielectric materials, such as those adopted by us here, can have small absorption. Among others, glass is an excellent example.

## DISCUSSION

To optically pull a particle effectively, one will have to redirect the incident light forward. Transverse isotropy, which forbids energy to propagate in the azimuthal direction, can regulate scattering to enhance the OPF. This would allow one to achieve the OPF at θ_0_ ≈ 30^o^ for micrometer-scale particles, which corresponds to a range of about ~0.4 to 0.7 cm. ARC can eliminate reflection. Cylindrical geometry, as enforced by Snell’s law, can regulate the light diffraction so that light can propagate along the edge of the pulling cone. Last, the excitation of the FWM can collimate the diffracted light through interference, which forces the scattered light to bend into the pulling cone. The dielectric cylinder and Bessel beam system can operate as an OTB with a macroscopic range. We stress that these mechanisms can also work separately, which will allow more flexibility in the design.

We predicted the OPF at θ_0_ = 1^o^ for an ARC-coated cylinder (*n*_p_ = 1.6) in water (*n*_b_ = 1.33). The cylinder’s diameter and length were *D* = 28 μm and *L* = 0.9 μm, respectively. The angle of 1^o^ was the smallest angle we attempted. We found no lower bound in converging angle other than θ_0_ = 0^o^. The search for an even smaller θ_0_ with OPF is limited by the computational resources available rather than by physical laws. Suppose that we generate a Bessel beam using an axicon; then, the range of the Bessel beam corresponding to θ_0_ = 1^o^ is estimated to be ~14.3 cm, which is also the range of the OTB. This range is unprecedented for the OPF. We note that an optical pushing force induced by a Bessel beam with a macroscopic range has already been demonstrated (*46*). Our work adds the function of pulling to the manipulation in the “macroscopic range.”

To pull a particle back to its source, mechanical stability in the transverse and the angular directions is also necessary. Here, by applying the force constant matrix approach (*69*), we explicitly verified the stability of the rotational and translational degrees of freedom for some of our structures exhibiting optical pulling. We note that for optical pulling in water, Brownian fluctuation may tilt or displace the particle relative to the beam. We show in section S4 that, for a microcylinder, the optical pulling can survive for some tilting and displacement and that Brownian fluctuations can destroy neither the OPF nor its stability. With all these, we successfully achieved macroscopically ranged OTB with good tolerance of the Brownian motion, half-cone angle, frequency bandwidth, and particle morphology.

Last but not least, in addition to the impedance-matched metamaterial discussed here, we are aware that metasurfaces are also a very tempting approach to induce OPF: Guided by the generalized Snell’s law, they can be designed to steer the incident light forward. However, there are also difficulties, such as the material absorption and the fabrication for the metasurfaces on a microparticle. These will be interesting topics for further research.

## MATERIALS AND METHODS

### Calculation of electromagnetic fields

All numerical calculations presented in this paper are full-wave electrodynamic calculations done by the commercial finite element package COMSOL Multiphysics, which provides an accurate solution to the Maxwell equations in the frequency domain. Two modules of the software were used, namely, the 2D axisymmetric radio frequency module and the 3D radio frequency module. For the transversely isotropic configuration, the axisymmetric module was adopted. By using symmetry, the axisymmetric model effectively reduced the simulation complexity from 3D to 2D. In addition to increased efficiency, it also allowed us to study much larger particles, up to tens of micrometers in diameter. When appropriate, the accuracy of the axisymmetric module was checked against the standard 3D radio frequency module and against the generalized Mie theory for spherical particles (*69*). In all cases, the agreement was remarkable. Nontransversely isotropic systems, such as the dielectric blocks illuminated by a Bessel beam, were treated by the standard 3D radio frequency module.

### Calculation of optical force

The time-averaged optical forces could be computed by integrating the time-averaged Maxwell stress tensor $\langle \overset{↔}{T}\rangle$ over a surface enclosing the particle

$$\langle F\rangle ={∯}_{S}\hat{n}\cdot \langle \overset{↔}{T}\rangle d\sigma$$ (12)

where

$$\langle \overset{↔}{T}\rangle =\frac{1}{2}\text{Re}[\varepsilon_{r}\varepsilon_{0}EE*+\mu_{0}HH*-\frac{1}{2}(\varepsilon_{r}\varepsilon_{0}E\cdot E*+\mu_{0}H\cdot H*)\overset{↔}{I}]$$ (13)

Here, the relative dielectric constant is given by

$$\varepsilon_{r}=\{\begin{array}{cc} 1, & \text{for air or vacuum}\\ 1.33^{2}, & \text{for water} \end{array}$$ (14)

**E** and **H** are the total electromagnetic fields and ε_0_ and μ_0_ are the permittivity and permeability of free space, respectively.

Optical forces for a lossless particle can also be computed from (*29*)

$$\begin{array}{c} \langle F_{z}\rangle ={\langle F_{z}\rangle }_{\text{interception}}+{\langle F_{z}\rangle }_{\text{recoil}}\\ =\;\text{cos}\;\theta_{0}\oint_{\text{Infinity}}g_{\text{sca}}(\theta )c\cdot \hat{n}d\sigma -\oint_{\text{Infinity}}g_{\text{sca}}(\theta )c\;\text{cos}\;\theta \cdot \hat{n}d\sigma \end{array}$$ (15)

where $g_{\text{sca}}=\;\text{Re}(E_{\text{sca}}\times H_{\text{sca}}^{*})/2c^{2}$ denotes the momentum density for the scattered wave. Our calculation mainly used Eq. 15 as it required less dense meshes to achieve convergence, and its results agreed remarkably with Eq. 12 when the mesh was sufficiently dense.

### Normalization of field intensity

A number of incident beams were adopted in this study. For Eqs. 4 to 6, the intensity at the beam center was normalized to 1 mW μm^−2^. This normalization could not be applied to the *m* = 0 azimuthally polarized Bessel beam given in Eq. 3 because it had a dark center along the beam axis. Thus, we normalized the intensity maximum of the beam to 1 mW μm^−2^. We note that 1 mW μm^−2^ was a modest intensity, and we were more concerned about the direction of the force than its exact magnitude.

## Supplementary Material

http://advances.sciencemag.org/cgi/content/full/5/3/eaau7814/DC1

Download PDF

## SUPPLEMENTARY MATERIALS

Supplementary material for this article is available at http://advances.sciencemag.org/cgi/content/full/5/3/eaau7814/DC1 Section S1. OPF acting on a spheroid Section S2. Small-angle approximation to the diameter associated with the FWM Section S3. Diffraction theory for slit and circular aperture to explain the negative recoil force induced by a pair of nearly forward propagating plane wave Section S4. Tolerance of the optical pulling on the misalignment of the particle due to Brownian motion Section S5. Tolerance of the optical pulling on the thickness and nonuniformity of the ARCs Fig. S1. OPF exerted on a spheroid by the *m* = 0 azimuthally polarized Bessel beam with θ_0_ = 30^o^. Fig. S2. Collimation of two nearly forward propagating plane waves through single-slit diffraction. Fig. S3. Collimation of two nearly forward propagating plane waves through diffraction through a circular aperture. Fig. S4. Robustness of OPF versus orientation. Fig. S5. Robustness of OPF versus thickness and nonuniformity of ARC.
