# Supplementary material for: Optical pulling at macroscopic distances
Source: Sci Adv. 2019 Mar 29;5(3):eaau7814. doi: 10.1126/sciadv.aau7814 (PMC6440754; doi:10.1126/sciadv.aau7814)
Supplement: http://advances.sciencemag.org/cgi/content/full/5/3/eaau7814/DC1 [file supp_5_3_eaau7814__index.html]

Science Advances | Science Advances

## Supplementary Materials

**This PDF file includes:**

- Section S1. OPF acting on a spheroid
- Section S2. Small-angle approximation to the diameter associated with the FWM
- Section S3. Diffraction theory for slit and circular aperture to explain the negative recoil force induced by a pair of nearly forward propagating plane wave
- Section S4. Tolerance of the optical pulling on the misalignment of the particle due to Brownian motion
- Section S5. Tolerance of the optical pulling on the thickness and nonuniformity of the ARCs
- Fig. S1. OPF exerted on a spheroid by the *m* = 0 azimuthally polarized Bessel beam with θ0 = 30o.
- Fig. S2. Collimation of two nearly forward propagating plane waves through single-slit diffraction.
- Fig. S3. Collimation of two nearly forward propagating plane waves through diffraction through a circular aperture.
- Fig. S4. Robustness of OPF versus orientation.
- Fig. S5. Robustness of OPF versus thickness and nonuniformity of ARC.

Download PDF

**Files in this Data Supplement:**

- Adobe PDF - aau7814\_SM.pdf
