# Supplementary material for: Optical pulling at macroscopic distances
Source: Sci Adv. 2019 Mar 29;5(3):eaau7814. doi: 10.1126/sciadv.aau7814 (PMC6440754; doi:10.1126/sciadv.aau7814)
Supplement: Download PDF [file aau7814_SM.pdf]

Science Advances
AAAS

advances.sciencemag.org/cgi/content/full/5/3/eaau7814/DC1

Supplementary Materials for

Optical pulling at macroscopic distances

Xiao Li, Jun Chen, Zhifang Lin, Jack Ng*

*Corresponding author. Email: jacktfng@hkbu.edu.hk

Published 29 March 2019, *Sci. Adv.* **5**, eaau7814 (2019)
DOI: 10.1126/sciadv.aau7814

This PDF file includes:

Section S1. OPF acting on a spheroid
Section S2. Small-angle approximation to the diameter associated with the FWM
Section S3. Diffraction theory for slit and circular aperture to explain the negative recoil force induced by a pair of nearly forward propagating plane wave
Section S4. Tolerance of the optical pulling on the misalignment of the particle due to Brownian motion
Section S5. Tolerance of the optical pulling on the thickness and nonuniformity of the ARCs
Fig. S1. OPF exerted on a spheroid by the $m = 0$ azimuthally polarized Bessel beam with $\theta_0 = 30^o$.
Fig. S2. Collimation of two nearly forward propagating plane waves through single-slit diffraction.
Fig. S3. Collimation of two nearly forward propagating plane waves through diffraction through a circular aperture.
Fig. S4. Robustness of OPF versus orientation.
Fig. S5. Robustness of OPF versus thickness and nonuniformity of ARC.

Section S1. OPF acting on a spheroid

The main text primarily focuses on the optical pulling force (OPF) acting on dielectric cylinders. The advantage of this is that anti-reflection coatings may be applied on their ends to boost the OPF. Nevertheless, other transversely isotropic objects, such as spheroids, also exhibit a strong pulling effect, as shown in fig. S1. The plot in fig. S1(B) shows the phase space region with OPF (blue), which is significantly larger than that of a bare cylinder of similar size and material composition. The OPF is quite strong and common at 30° for a spheroid.

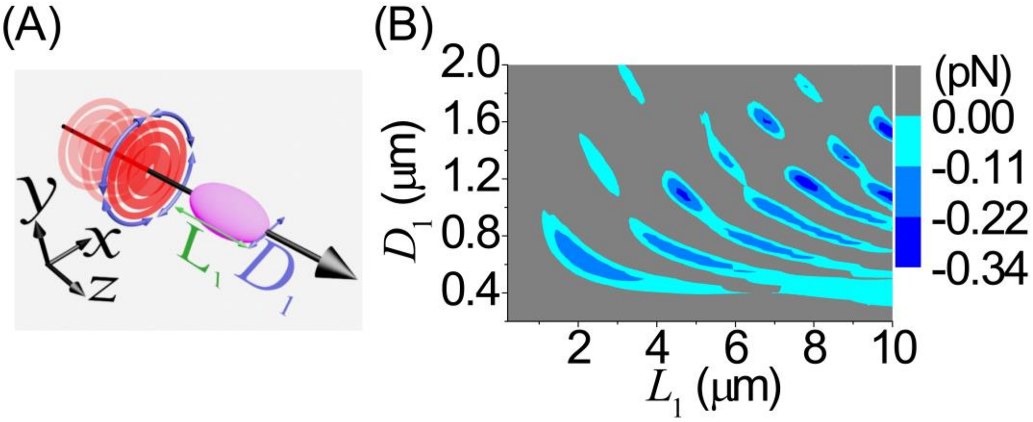

Fig. S1. OPF exerted on a spheroid by the *m* = 0 azimuthally polarized Bessel beam with $\theta_0 = 30^o$. (**A**) A schematic illustration of the geometry. (**B**) The OPF versus length L_1 and diameter D_1.

Section S2. Small-angle approximation to the diameter associated with the FWM

The micro-cylinder we considered can be viewed as a waveguide. We are only interested in the fundamental waveguide modes, which separate into the transverse magnetic (TM) (H_ϕ, E_r, E_z) and transverse electric (TE) (E_ϕ, H_r, H_z) groups. The cutoff condition for the TM group can be obtained by solving [68]

$$\frac{J_1(u)}{J_0(u)} = -\frac{\varepsilon_b u}{\varepsilon_p w}\frac{K_1(w)}{K_0(w)}, \qquad (.1)$$

while the corresponding equation for the TE set is

$$\frac{J_1(u)}{J_0(u)} = -\frac{u}{w}\frac{K_1(w)}{K_0(w)}, \qquad (.2)$$

where $J_n(x)$ is the Bessel function of the first kind, $K_n(x)$ represents the modified Bessel function of the second kind, and ε_p and ε_b are the permittivity inside and outside the dielectric waveguide, respectively. For a waveguide with radius a, one can define $u^2 = (k_p^2 - \beta^2)a^2$ and $w^2 = (\beta^2 - k_b^2)a^2$, where β is the propagating wavenumber inside the waveguide, and k_p and k_b are the wavenumbers inside and outside the waveguide, respectively.

For $\beta = k_p \cos\theta_p$, one has $u = k_p \sin\theta_p a$ and $w = \sqrt{k_p^2 - k_b^2 - k_p^2(\sin\theta_p)^2}\,a$. As $\theta_p \to 0$, u vanishes. Consequently, the solutions to (.1) or (.2) are given by $J_1(u) = 0$, which, for small θ_p, can be solved to give

$$a(\theta_p) = z_1 / k_p \theta_p, \qquad (.3)$$

where z_1 fulfills $J_1(z_1) = 0$. Applying Snell's law for a small angle gives $\theta_p = n_b \theta_0 / n_p$, the diameter for the fundamental mode is given by

$$D(\theta_0) = 2z_1 / k_b \theta_0 \qquad (.4)$$

Section S3. Diffraction theory for slit and circular aperture to explain the negative recoil force induced by a pair of nearly forward propagating plane wave

The main text discussed how interference between different Fourier components of the diffracted field can lead to a negative recoil force. Here we show that this phenomenon does not solely occur for cylinders, but also for a long slit.

The scalar theory of diffraction by a single long slit is discussed in a number of textbooks. Here, we recap its essence and explain how it could lead to a negative recoil force. The diffracted field from the slit due to uniform illumination by a pair of plane waves tilted at an angle $\pm\theta_0$ perpendicular to the slit is given by

$$E_{\text{diff},\pm}(\theta) = E_0 \text{sinc}\left(\frac{kb}{2} (\sin\theta \pm \sin\theta_0) \right), \qquad (.5)$$

where $\text{sinc}(x) = \sin x / x$. Eq. (.5) shows that the angular distribution of the diffraction field is centered at the direction $\theta = +\theta_0$ for $E_{\text{diff},+}$ and $\theta = -\theta_0$ for $E_{\text{diff},-}$. When the first odd mode of the slit is excited, the slit width is given by

$$b(\theta_0) = \frac{2\pi}{k \sin\theta_0} \qquad (.6)$$

and $E_{\text{diff},+}$ and $E_{\text{diff},-}$ are out of phase at $\theta = 0^{\circ}$. The interference between the diffracted waves constructive when $\theta \approx 0^{\circ}$, which shifts the peak from $\theta = \pm\theta_0$ into the pulling cone defined by $|\theta| < \theta_0$, as shown in fig. S2(B) for $\theta_0 = 2^{\circ}$.

For a cylinder with diameter $D=14\mu\text{m}$ and length $L=0.9\mu\text{m}$ illuminated by the incident wave in Eq. (1) with $\theta_0 = 2^\text{o}$ in the main text, the scattering amplitude is plotted in Figure 3(I). In fig. S3(A) where there is only one incident plane wave, the scattering amplitude is strongest at $\theta = \theta_0 = 2^\text{o}$. Similarly, fig. S3(B) also shows the scattering amplitude for the other plane wave, which is strongest at $\theta = -2^\text{o}$. When both plane waves are present and interfere with each other, the region with strong scattering amplitude is clearly pulled into the pulling cone.

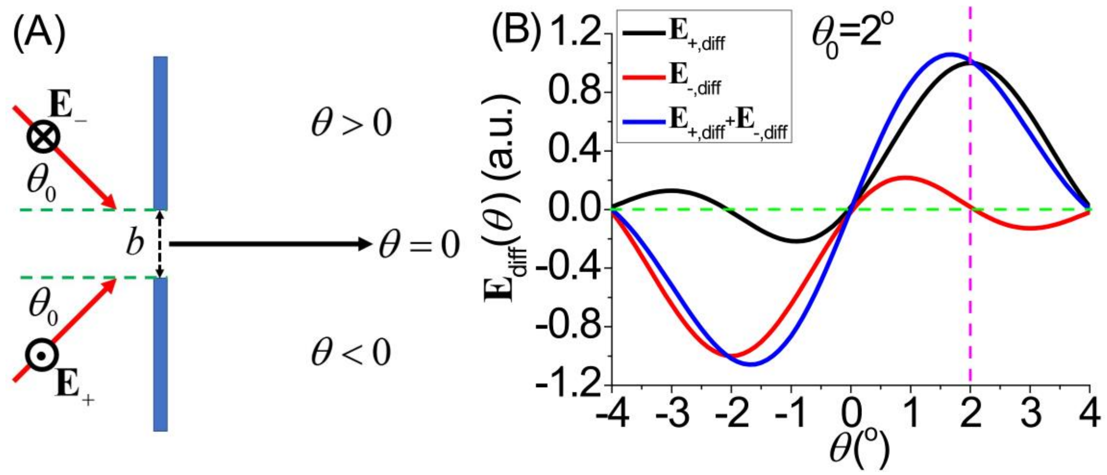

Fig. S2. Collimation of two nearly forward propagating plane waves through single-slit diffraction. (**A**) Illustration of a single slit diffraction when two out-of-phase incident plane waves illuminate on it at angle θ_0. The slit has a width of $b(\theta_0)$. (**B**) Diffracted fields versus θ for $\mathbf{E}_+$, $\mathbf{E}_-$, and $\mathbf{E}_+ + \mathbf{E}_-$.

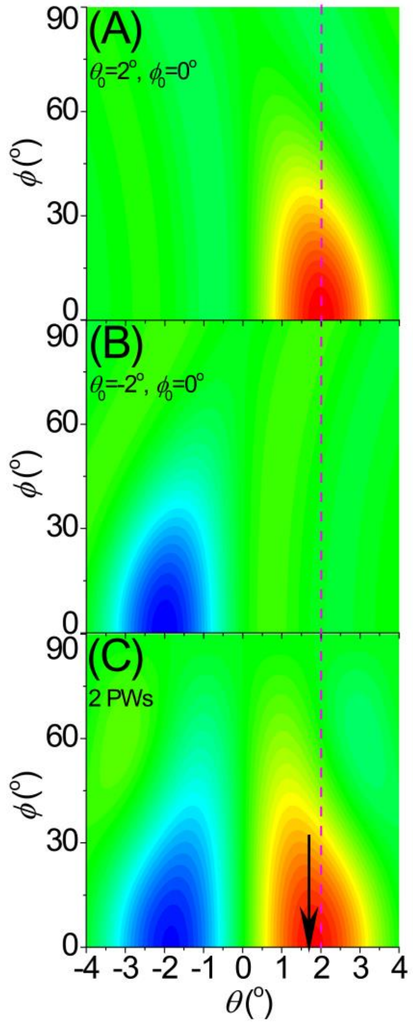

Fig. S3. Collimation of two nearly forward propagating plane waves through diffraction through a circular aperture. (**A**) One plane wave propagating along $(\theta_0 = 2^{\circ}, \phi_0 = 0^{\circ})$. (**B**) One out-of-phase plane wave propagating along $(\theta_0 = -2^{\circ}, \phi_0 = 0^{\circ})$. (**C**) Two out-of-phase plane waves propagating along $(\theta_0 = 2^{\circ}, \phi_0 = 0^{\circ})$ and $(\theta_0 = -2^{\circ}, \phi_0 = 0^{\circ})$. Here we define $\theta \in [-\pi, \pi)$ and $\phi \in [0, \pi)$. The peak direction with maximum scattering is shifted from $\theta_0 = \pm 2^{\circ}$ in (A) and (B) to within $|\theta| < 2^{\circ}$ in (C).

Section S4. Tolerance of the optical pulling on the misalignment of the particle due to Brownian motion

In typical optical trapping experiment, the Brownian fluctuation can tilt the particle such that its axes is misaligned with that of light. In this section, we show that this mis-alignment of the particle due to Brownian fluctuation is small, and optical pulling force survives with this small fluctuation.

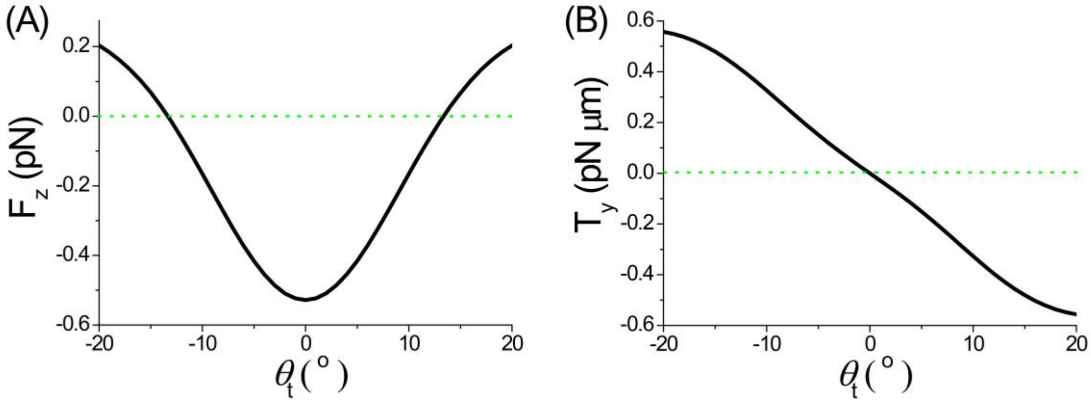

Fig. S4. Robustness of OPF versus orientation. Optical pulling force F_z (A) and restoring torque T_y (B) versus the tilted angle θ_t around y-axis. An azimuthally polarized Bessel beam with a central ring power of 1 mW and a half cone angle 30° illuminates the cylinder of diameter 600nm and length 1500nm.

It is well-known that Brownian fluctuations are typically small for optically trapped micro-particles even at a small laser power on the order of mW. And in the main text, when considering the mechanical stability of the cylindrical particle, the fact that the particle is stable already implied the survival of the pulling force for small displacement or rotation.

To make it more quantitative, we explicitly plotted the pulling force (fig. S4(A)) and the restoring torque (fig. S4(B)) versus the tilted angle θ_t, where a dielectric cylinder in water ($n_p / n_w = 1.6/1.33$) is illuminated by an azimuthally polarized Bessel beam with a half cone angle of 30°. Since the system is transversely isotropic, we may consider only the rotation around the y-axis. The situations for other axes are identical by symmetry.

From fig. S4(A), it is clear that the pulling force survives for a mis-alignment as big as $-13^o < \theta_t < +13^o$. We then argue that the Brownian motion induced misalignment is always safely within this range even for small laser power.

By equipartition theorem, the average thermal energy for rotation is $k_B T / 2$. While the restoring potential energy for rotation within the linear regime, i.e. $-13^o < \theta_t < +13^o$ (see fig. S4(B)), is given by $U = k_{\theta_t} \theta_t^2 / 2$ where $k_{\theta_t} \approx 1.9$ pN μm / rad at 1 mW of laser power for the central ring of the Bessel beam. Accordingly, an energy of $k_B T / 2$ at room temperature (300 K) will induce a mis-alignment of only ~2.6 Degrees. On the other hand, as shown in fig. S4(A), the force remained negative for $-13^o < \theta_t < +13^o$. We further note that even for a fluctuation of ten times the average thermal energy ($10 \times k_B T / 2$), which is highly unlikely to occur, the induced mis-alignment is still ~8.5 degrees, safely within the pulling range of $-13^o < \theta_t < +13^o$. In fact, the rotational trap depth is given by

$$\int_{0^o}^{13^o} \tau_y d\theta_t = 11.7 k_B T \ ,$$

which is more than 23 times the average thermal energy in room temperature.

Last but not least, the calculated restoring force constant for the particle displacement in the transverse plane is $2.47 \ \ \text{pN} / \mu\text{m}$. Accordingly, by equipartition theorem, the corresponding average thermal fluctuation will be ~40 nm, which is small compared to wavelength, and therefore it does not affect the optical pulling.

In short, although Brownian motion can be strong for optically trapped nano-particles, for the micro-particle discussed in the manuscript, Brownian motion is not a major concern. Finally, we remark that our approach also works in vacuum, where Brownian motion is absent.

Section S5. Tolerance of the optical pulling on the thickness and nonuniformity of the ARCs

The thickness and non-uniformity of the anti-refection coatings have a high degree of tolerance in many applications. Our calculations below show this is also the case for optical pulling. A 900 nm long dielectric cylinder (see inset for an illustration) in water with a refractive index of 1.6 is illuminated by an azimuthally polarized Bessel beam (Eq. (1) of the main text) of a half cone angle of 10°. The ideal thickness of the anti-reflection coating is d=92 nm.

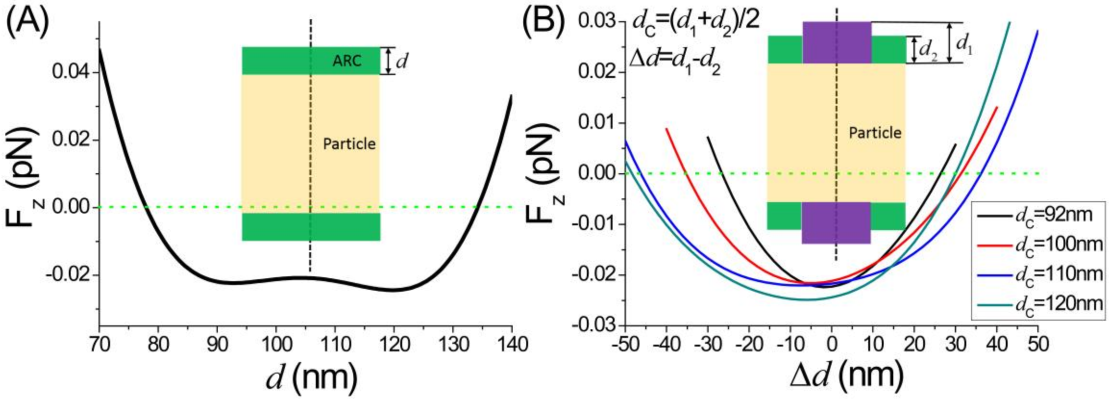

Fig. S5. Robustness of OPF versus thickness and nonuniformity of ARC. Optical pulling force (OPF) F_z exerted on an ARC-coated cylinder versus the ARC thickness d (**A**) and ARC thickness difference Δd (**B**). An azimuthally polarized Bessel beam with a half cone angle of 10° is illuminating the cylinder with diameter $D(10^o)$ and length 900nm. In (A), OPF survives for d=78-133nm, while the theoretical optimal thickness is 92nm. In (B), the thickness of the ARC has a step. The thickness for the center part, $r< D/4$, is d_1, while that of the outer part, $r> D/4$ is d_2. Here the thickness difference was defined as $\Delta d=d_1-d_2$. Different "mean" thicknesses, defined as $d_C=(d_1+d_2)/2$, are considered in (B). The OPF is plotted against Δd.

Figure S5(A) shows pulling force versus coating thickness d. The pulling force is basically unaffected for d=85-125 nm, and it survives for d=78-133 nm. We note that as illustrated in fig. S5(A), the pulling is less affected when the thickness is slightly thicker than its ideal value of 92 nm, but more affected when the thickness is slightly thinner.

Figure S5(B) shows pulling force versus non-uniform thickness d_1 (inner part thickness) and d_2 (outer part thickness), see inset for a schematic illustration. Different colors represent different "mean" thickness $d_C = (d_1 + d_2)/2$. To see how non-uniform thicknesses affect the pulling force, we calculate for non-uniform coatings with thickness d_1 for $r< D/4$ (purple) and d_2 for $D/4$ (green), i.e. the inner and outer part of the coating have different thicknesses. For $d_C=(d_1+d_2)/2$ being 92nm, 100nm, 110nmm, or 120nm, the pulling force versus non-uniformity in thickness $\Delta d=d_1-d_2$ is plotted. The pulling force clearly survived for a wide range of Δd, on the order of tens of nm. In particular, for $d_C = 110$ nm, the pulling force survives for $-46\text{nm} < \Delta d < 36\text{nm}$. In other words, optical pulling survives for non-uniformity in thickness, with a tolerance of several tens of nm.
